# The erratic mitochondrial clock: variations of mutation rate, not population size, affect mtDNA diversity across birds and mammals

**DOI:** 10.1186/1471-2148-9-54

**Published:** 2009-03-10

**Authors:** Benoit Nabholz, Sylvain Glémin, Nicolas Galtier

**Affiliations:** 1 Université Montpellier 2 CNRS UMR 5554 – Institut des Sciences de l'Evolution Place E. Bataillon – CC064, 34095 Montpellier, France

## Abstract

**Background:**

During the last ten years, major advances have been made in characterizing and understanding the evolution of mitochondrial DNA, the most popular marker of molecular biodiversity. Several important results were recently reported using mammals as model organisms, including (i) the absence of relationship between mitochondrial DNA diversity and life-history or ecological variables, (ii) the absence of prominent adaptive selection, contrary to what was found in invertebrates, and (iii) the unexpectedly large variation in neutral substitution rate among lineages, revealing a possible link with species maximal longevity. We propose to challenge these results thanks to the bird/mammal comparison. Direct estimates of population size are available in birds, and this group presents striking life-history trait differences with mammals (higher mass-specific metabolic rate and longevity). These properties make birds the ideal model to directly test for population size effects, and to discriminate between competing hypotheses about the causes of substitution rate variation.

**Results:**

A phylogenetic analysis of cytochrome *b *third-codon position confirms that the mitochondrial DNA mutation rate is quite variable in birds, passerines being the fastest evolving order. On average, mitochondrial DNA evolves slower in birds than in mammals of similar body size. This result is in agreement with the longevity hypothesis, and contradicts the hypothesis of a metabolic rate-dependent mutation rate. Birds show no footprint of adaptive selection on cytochrome *b *evolutionary patterns, but no link between direct estimates of population size and cytochrome *b *diversity. The mutation rate is the best predictor we have of within-species mitochondrial diversity in birds. It partly explains the differences in mitochondrial DNA diversity patterns observed between mammals and birds, previously interpreted as reflecting Hill-Robertson interferences with the W chromosome.

**Conclusion:**

Mitochondrial DNA diversity patterns in birds are strongly influenced by the wide, unexpected variation of mutation rate across species. From a fundamental point of view, these results are strongly consistent with a relationship between species maximal longevity and mitochondrial mutation rate, in agreement with the mitochondrial theory of ageing. Form an applied point of view, this study reinforces and extends the message of caution previously expressed for mammals: mitochondrial data tell nothing about species population sizes, and strongly depart the molecular clock assumption.

## Background

Animal mitochondrial DNA (mtDNA) evolution contrasts with nuclear evolution. Mitochondrial and nuclear genomes differ in many ways, such as total length, ploidy level, mode of inheritance, recombination rate, presence of introns, percentage of non-coding DNA, effective population size, and repair mechanisms, *e.g*. [1,2]. Among these particularities, hypermutability is one of the most striking features of animal mitochondria: the mtDNA mutation rate is typically one order of magnitude higher than the nuclear one [1,3,4]. The question of the origin and evolution of such high mutation rates is still open and debated [5,6]; and see [7,8] for exceptions.

This high mutation rate is one of the reasons why mtDNA is a very popular marker for biodiversity studies. For example, it has been massively used to investigate intraspecific to intra-ordinal evolutionary relationship, and disentangle rapid speciation events in phylogenetic studies (*e.g*. see [9,10] for a comparison of nuclear and mitochondrial markers). In recent years, mtDNA has been used to identify species using a standardized portion of the cytochrome oxydase I gene (COI), according to the so-called DNA barcoding approach [11]. The high mtDNA mutation rate, however, is also the source of frequent homoplasy, *i.e*., phylogenetic incongruence between sites of the molecule because of multiple mutations at the same site. Homoplasy complicates the use of mtDNA in phylogenetic [9,10,12] and even population genetic [13] studies.

We recently reported several results related to mtDNA mutation dynamics in mammals. First, we showed that mtDNA substitution rates are extremely variable between species: they differ by two orders of magnitude between slow-evolving and fast-evolving mammalian lineages [14], definitively rejecting the famous "2% per site per million year" calibration, which should not be generally trusted. We proposed that mutation rate variations are possibly linked to species longevity through the action of natural selection: too high a mutation rate would be deleterious in long-lived mammals because it could result in premature aging due to the accumulation of somatic mtDNA mutations [14], see also [15-17]. Secondly, we found that within-species cytochrome *b *(cytb) nucleotide diversity is correlated with the mitochondrial mutation rate, as expected, but not with any life-history or ecological variable potentially related to population size, including body size, geographic range and conservation status [18]. This surprising pattern was apparently not explained by selective effects [18] (see also [19]), contrary to results obtained at the Metazoa level [20], especially invertebrates [21]. We therefore proposed that the lack of correlation between mtDNA diversity and potential indicators of population size in mammals is due to strong demographic stochasticity. At any rate, the mutation rate is the best predictor we have of mitochondrial genetic diversity across mammalian species. Overall, these results highlight the importance of mutation rate variations in shaping mtDNA biodiversity patterns in mammals, and suggest that mutational effects should be carefully taken into account when analysing such data.

In this study, we extend our analysis of the evolutionary dynamics of mtDNA through the bird/mammal comparison. Our objective is dual. First, we want to check whether the mammalian results are specific to this group, or have any degree of generality. Secondly, we want to make use of the genetical and physiological specificities of birds to test various evolutionary hypothesis raised by previous studies. Birds are the perfect candidate to challenge the results obtained in mammals: like mammals, they include charismatic, well studied species, in which a large amount of genetic and biological data are available. Being relatively large, warm blooded vertebrates, birds are comparable to mammals in terms of physiology, ecology, and life-history. Birds, furthermore, show a number of peculiarities potentially relevant to mitochondrial evolution and diversity.

The first reason why birds are appropriate for a comparative approach is the availability of direct population size estimates obtained through global population surveys, especially for North American species [22]. Birds therefore provide the opportunity to directly test the relationship between population size and mitochondrial diversity, whereas in mammals we had to rely on ecological and life history traits, plausibly, but only supposedly, correlated to population size. We will therefore check whether the lack of relationship between mtDNA diversity and species abundance we reported in mammals is confirmed in birds, or was due to inappropriate measure of the effective population size.

Birds, secondly, present a genetic peculiarity: female is the heterogametic sex. Because it is strictly maternally transmitted, the avian mitochondrial genome is thus in full genetic linkage with the female-specific W chromosome [23]. Berlin *et al*. [24] proposed that the mtDNA diversity could therefore be reduced by Hill-Robertson interference: selective effects applying to loci linked to the W will affect mtDNA through hitch-hiking. This clever hypothesis has provoked some comments and reactions [24-27]. Hickey [26] questioned one of the most important arguments of Berlin *et al*. [24], namely the lower synonymous diversity in bird than in mammal mtDNA, arguing that mutation rate could be a confounding effect. An accurate estimation of mtDNA substitution rate variations in birds appears necessary to correctly interpret the patterns of mtDNA diversity in the bird/mammal comparison.

Birds, finally, have quite high metabolic rates – 1.5 to 2.5 times higher than mammals of similar sizes [28] -probably because of the high energetic demand of the flying locomotion. Paradoxically, birds species are strikingly long-lived as compared to their mammalian counterparts; on average, birds live three times longer than mammals of similar sizes [28,29]. Birds can be characterized as long-lived homeotherms, a specificity they share with bats [30]. These peculiarities are useful to understand further the impact of metabolic rate and life-span on mtDNA substitution rates.

Three main hypotheses have been proposed to explain mtDNA substitution rate variations in mammals: the generation time hypothesis [30-33], the metabolic rate hypothesis [34,35], and the longevity hypothesis [14-17], presented above. The classical approach to test these hypotheses in a comparative framework is to correlate substitution rate variations with the relevant life history traits [14,17,33]: female sexual maturity, basal mass-specific metabolic rate (or body mass, taken as a proxy), and maximum longevity. However, these life-history traits are strongly correlated with each other, so that the respective contributions of the three variables are difficult to disentangle. The contrast between the avian and mammalian physiologies provides a unique opportunity to discriminate between two of these competing models, namely the longevity and the metabolic rate hypotheses. According to the longevity hypothesis, birds should present, on average, lower mtDNA neutral substitution rates than mammals, while the reverse pattern is expected under the metabolic rate hypothesis.

To test these predictions and challenge the results we obtained in mammals, we propose to accurately estimate the lineage-specific neutral substitution rate variation of mtDNA in birds using the phylogenetic framework developed by [14]. Using this dataset, we want to check the rough 2%/site/Myr calibration. This calibration, originally estimated with an RFLP analysis on mammals by Brown *et al*. [3], was generalized to birds by a study in geese [36]. This calibration rapidly became a standard in ornithology studies, probably because of the scarcity of the fossil record (see [37,38] and [39] for a review). Recently, the reliability of this calibration was debated in various studies either supporting [40-42] or rejecting it [39,43]. We will also ask which of metabolic rate and longevity is the best predictor of mutation rate variations in warm-blooded vertebrates. Finally, we will estimate the mitochondrial genetic diversity (synonymous and non-synonymous) on a wide taxonomic range in birds to test for the neutrality assumption and investigate the link between population size, mutation rate, genetic hitch-hiking, and mtDNA diversity.

## Results

### Substitution rate variation and its determinants

To infer the substitution rate variation of a large number of bird species, we need to estimate divergence dates, and the numbers of nucleotide substitutions which occurred during these divergences (branch lengths). The divergence dates are obtained thanks to paleontologic calibration points, which are mostly available for relatively old divergences. Nucleotide substitution numbers, however, must be estimated using little-divergent sequences because of mutational saturation. To resolve this methodological problem, we take advantage of the decoupled non-synonymous (*i.e*. amino acids) and synonymous evolutionary dynamics. We introduced a 3-step method [14], in which we first define groups of sequences showing limited divergences, then estimate the relative species-specific substitution rate at third codon positions within groups, and finally assign a divergence for each of these groups using amino acid sequences. At step 2 and 3, molecular and fossil information are combined thanks to a sophisticated Bayesian method [44].

**Figure 1:**
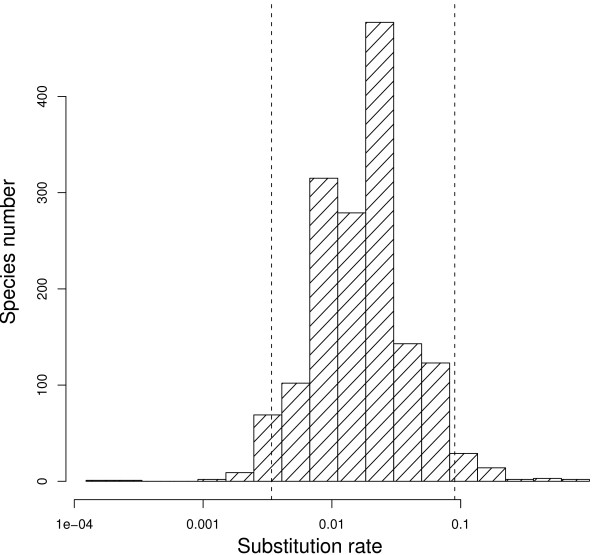
**Distribution of cytochrome b neutral substitution rate in 1,562 bird species**. Substitution rates are log transformed and are in unit of substitution per third codon position per million years.

**Figure 2:**
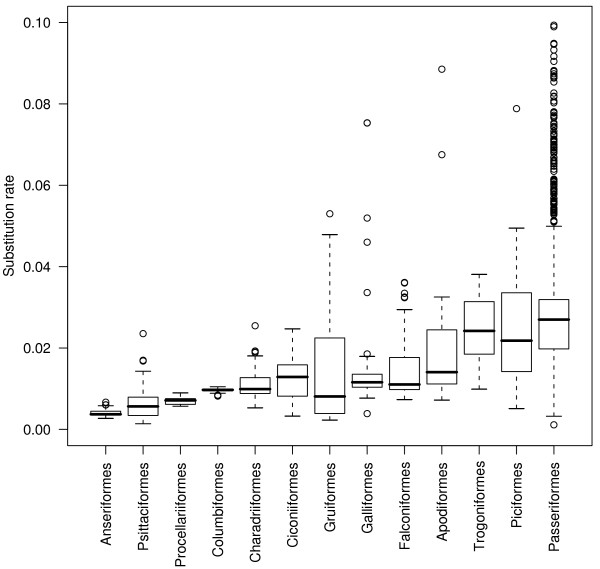
**Within-order distribution of the cytochrome b third codon position substitution rate**. Substitution rates are in unit of substitution per third codon position per million year. Orders represented by more than 20 species are shown. Values above 0.1 substitution/site/Myrs years are not shown.

**Figure 3:**
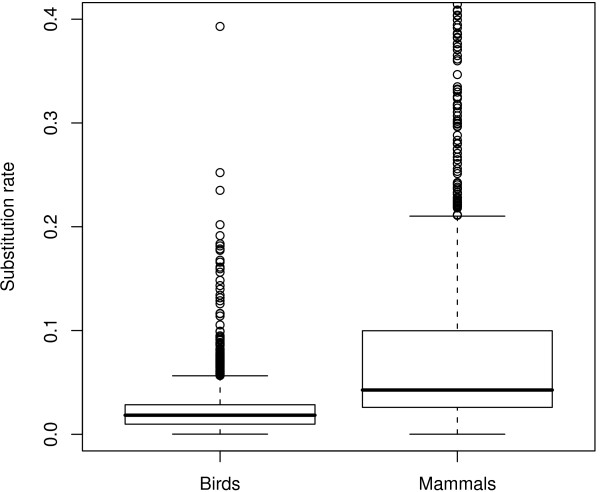
**Distribution of cytochrome b neutral substitution rate in 1,696 mammalian species (Data from **[14]**) and 1,571 birds species (this study)**. Substitution rates are in unit of substitution per third codon position per million years. Values above 0.4 substitution/site/Myrs years are not shown.

Using this procedure, we analysed a cytb dataset of 1571 species, which represent ~15% of the total living species of birds. The average cytb third codon position substitution rate was 0.027 substitution/site/Myr, and the median was 0.018 substitution/site/Myr. These substitution rates are estimated per lineage, per third-codons position. To make them roughly comparable to the popular 2% of divergence per site per Myr, these numbers must be multiplied by two (to reflect a divergence between two species) and divided by three (assuming that only third codon positions are variable). Doing this would yield an average divergence of 1.8% per Myr (median: 1.2% per Myr), which is slightly less than the popular 2%. The distribution across species shows a large variation: the 2.5% and 97.5% percentiles are 0.003 and 0.090 substitution/site/Myr, respectively (Figure 1). Thus, even if we consider the 5% most extreme substitution rate values as outliers, the cytb third codon position substitution rate shows a thirty-fold range of variation across bird lineages. We quantified the effect of taxonomy on substitution rate variation using a hierarchical ANOVA considering super-order, order, family and genus with random effects. The order level explains 46% (63% in simple one-way ANOVA) of the variance in substitution rates across species. Considering only order with more than 20 species, Passerines are the fastest evolving order (0.038 ± 0.055 substitution/site/Myr) and Anseriformes the slowest one (0.004 ± 0.0008 substitution/site/Myr, see Figure 2). Compared with mammals, birds globally evolve 3.7 times slower on average (mammalian average: 0.098 substitution/site/Myr, Nabhloz *et al*. [14]), and show a range of variation less extreme (coefficient of variation: 1,58 for birds, 1,72 for mammals, Figure 3).

**Figure 4:**
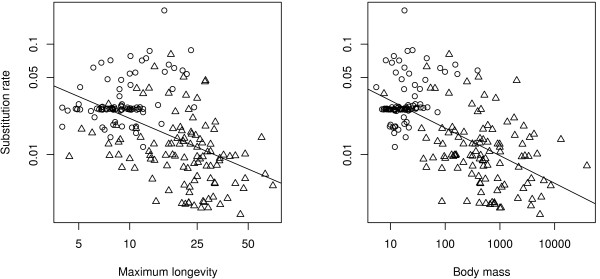
**Relationship between substitution rate and life-history traits**. a) Relationship with body mass (log transformed and in grams), b) Relationship with maximum longevity (log transformed and in years). Circles are for passerine birds (n = 88), triangles for non-passerine birds (n = 108). Substitution rates are log transformed and are in unit of substitution per third codon position per million years.

We correlated the species-specific substitution rate to life-history traits in order to discriminate between the competing explanatory hypotheses. A smaller amount of life-history data are available in birds than in mammals, especially for female sexual maturity, which is documented in 30 species only. We thus focused on the effects of body mass and maximum longevity using the 196 species in which these two variables are available. In agreement with both the metabolism and the longevity hypotheses, the two life-history traits are negatively linked to the substitution rate. Body mass appears to be a better predictor of the substitution rate (R^2 ^= 0.39, *p *< 0.001, n = 196) than maximum longevity, which also shows a strong level of correlation (R^2 ^= 0.25, *p *< 0.001, n = 196, Figure 4, Table 1).

**Table 1:** Effects of life-history variables on mtDNA substitution rate in bird species without (A) and with phylogenetic control (B).

Model	slope	R^2^	*p*1	*p*2
(A) Without Phylogenetic control				
Body mass	-0.25	0.39	<**0.01**	
Maximum longevity	-0.66	0.25	<**0.01**	
Body mass + Maximum longevity		0.39	<**0.01**	0.42

(B) With Phylogenetic control				
Body mass	-0.22	0.38	<**0.01**	
Maximum longevity	-0.46	0.19	<**0.01**	
Body mass + Maximum longevity		0.38	<**0.01**	0.89

The distribution of (log-transformed) maximum longevity is strongly bimodal in birds, passerines being substantially more short-lived than other birds (see Additional file 1). We separated passerines from non-passerine and re-performed the same analyses in the two subsets separately. The results are shown in Table 2. Surprisingly, the third-codon position substitution rate is positively correlated to body mass and maximum longevity in passerines birds. In non-passerines birds, results are similar to the global analysis: body mass best explains substitution rate variations, and removes the effect of longevity in a two-way ANOVA (Table 2). This result is not consistent with mammals [14].

**Table 2:** Effects of life-history variables on mtDNA substitution rate in passerines (n = 88) vs. other bird species (n = 108) without (A) and with phylogenetic control (B).

	Passerines				Non-passerines			
Model	slope	R^2^	*p*1	*p*2	slope	R^2^	*p*1	*p*2
(A) Without Phylogenetic control								
Body mass	0.22	0.11	<**0.01**		-0.17	0.12	<**0.01**	
Maximum longevity	0.48	0.15	<**0.01**		-0.41	0.09	<**0.01**	
Body mass + Maximum longevity		0.17	0.14	**0.02**		0.13	**0.01**	0.13

(B) With Phylogenetic control								
Body mass	0.22	0.12	<**0.01**		-0.11	0.05	**0.02**	
Maximum longevity	0.4	0.11	<**0.01**		-0.28	0.05	**0.03**	
Body mass + Maximum longevity		0.16	**0.04**	0.05		0.06	0.17	0.22

In order to check whether these results were robust to the removal of phylogenetic effects, we applied the phylogenetic contrast method [45] to regress phylogeny out of the analyses. In this analysis, we used the chronogramme obtained with amino-acid cytochrome *b *sequences. The phylogenetic control did not qualitatively change the results although most p-values were decreased (Table 1, Table 2).

**Figure 5:**
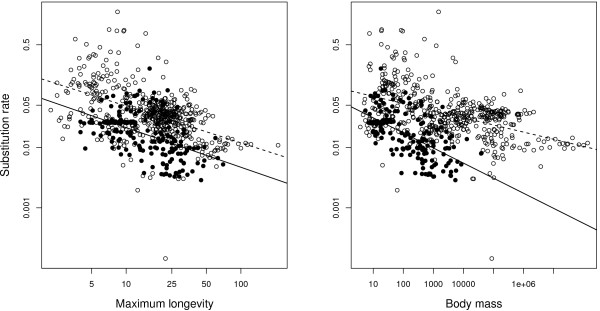
**Relationship between substitution rate and life-history traits**. a) Body mass (log transformed and in grams), b) Maximum longevity (log transformed and in years). Open circles and dotted regression line are for mammals (n = 500), close circles and solid regression line for birds (n = 196). Substitution rates are log transformed and are in unit of substitution per third codon position per million years.

Finally, we jointly analysed the mammalian and bird datasets (Table 3, Figure 5), including class (birds *vs*. mammals) as an explanatory variable. The two-factor model (class + life history trait) revealed that the bird *vs*. mammal status has a strong effect on mtDNA substitution rate when body mass is taken into account and a significant but weaker effect as far as longevity is concerned (Table 3). There is, moreover, a significant interaction between body mass and class (*p *< 0.001), but not between longevity and class (*p *= 0.65).

**Table 3:** Effects of life-history variables and bird (n = 196) vs. mammal (n = 500) status (referred as Class) on mtDNA substitution rate.

Model	Variable	Effect	t value	*P*
(A) Body mass + Class				
	Body mass	-0.25	-8.9	<**0.01**
	Class (Mammals)	0.22	3.1	<**0.01**
	Body mass: Class (Mammals)	0.13	4.4	<**0.01**

(B) Maximum longevity + Class				
	Maximum longevity	-0.65	-9.4	<**0.01**
	Class (Mammals)	0.31	2.5	**0.01**
	Maximum longevity: Class (Mammals)	0.04	0.5	0.65

### Determination of mtDNA diversity in birds

Bird cytb polymorphism sequence datasets were retrieved from the Polymorphix database [46]. The synonymous (*π*_*s*_) and non-synonymous (*π*_*n*_) levels of diversity were computed for the 147 species in which more than four sequences were available. The mean per-site synonymous nucleotide diversity was 0.040 ± 0.041. The most variable species was Shelley's Greenbul (*Andropadus masukuensis*, Pycnonotidae, Passeriformes, *π*_*s *_= 0.170), and two species showed no synonymous variations (Arabian Bustard, *Ardeotis arabs*, Otididae, Gruiformes, and Snowy owl, *Bubo scandiacus*, Strigidae, Strigiformes).

**Figure 6:**
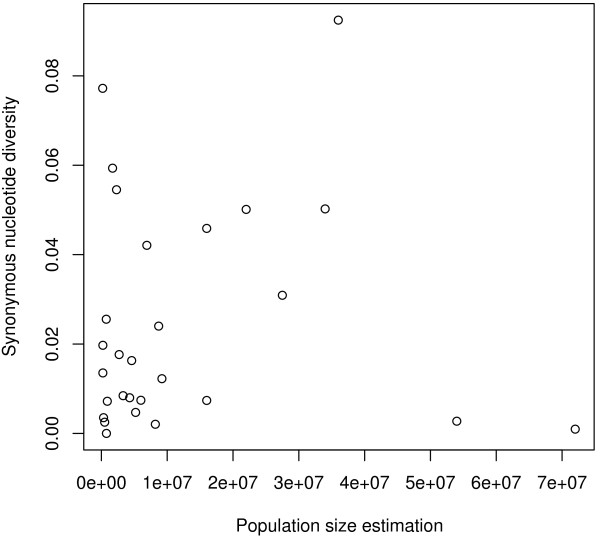
**mtDNA synonymous diversity (*π*_*s*_) vs. direct population size estimate in birds (n = 28)**. mtDNA synonymous diversity (*π*_*s*_) *vs*. direct population size estimate in birds (n = 28).

To test the relationship between population size and mtDNA diversity, we first correlated nucleotide polymorphism and body mass, taken as a proxy of population size, as in Nabholz *et al*. [18] and Berlin *et al*. [24]. *π*_*s *_and body mass were negatively correlated but, contrary to Berlin *et al*. [24], this relationship was not significant (R^2 ^= 0.019, *p *= 0.30, n = 56). Secondly, we applied a more direct analysis using population size estimates for North American breeding birds through the North American Landbird Conservation Plan [22]. Such data were available for 28 strictly North American species of our data set. We found no correlation between direct population size estimate and cytb synonymous diversity (Figure 6, t = 0.031, *p *= 0.81, Kendall's test). These results confirm those obtained in mammals [18], here using direct estimations of population sizes instead of life-history and ecological proxies. The dataset, however, is much smaller in the present analysis.

We tested the taxonomic effect on *π*_*s *_using one-way ANOVAs on order and family level. We found that neither the family nor the order level had a significant effect. The sole taxonomic variable having a significant effect on mtDNA diversity is the passerine/non-passerine status, passerines being more diverse than other birds (passerines: n = 79, non-passerines: n = 68, R^2 ^= 0.03, p < 0.05).

**Figure 7:**
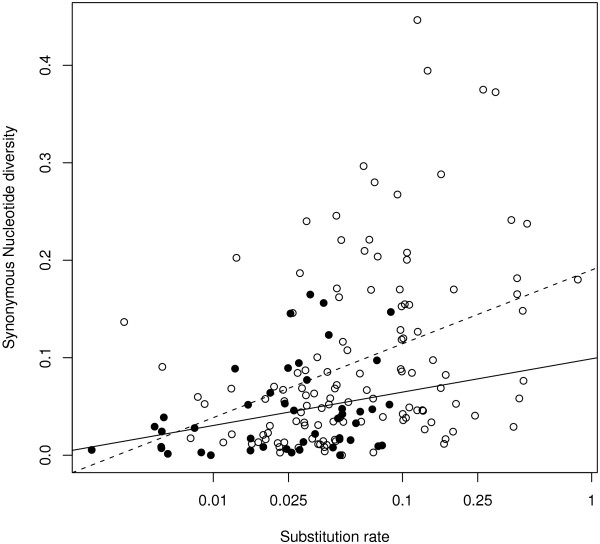
**mtDNA synonymous diversity (*π*_*s*_) and substitution rate**. Open circles and dotted regression line are for mammals (n = 123), close circles and solid regression line for birds (n = 46). Substitution rates are in log-transformed and are in unit of substitution per third codon position per million years.

We compared the distribution of *π*_*s *_between birds and mammals. Similarly to Berlin *et al*. [24], we found that birds have a lower average synonymous diversity than mammals (n = 169, R^2 ^= 0.09, p < 0.001, Additional file 2a), but this difference is largely reduced when we controlled for the mutation rate (previously estimated above after control, R^2 ^= 0.03, p = 0.032, Additional file 2b). Interestingly, we detected no significant difference of allozyme heterozygosity between mammals (average H = 0.046 ± 0.033) and birds (0.053 ± 0.028) using the Nevo *et al*. [47] data set (birds: n = 42, mammals: n = 164, R^2 ^= 0.01, p = 0.08). As in mammals, we found a positive and significant correlation between species-specific cytb third-codon substitution rate and cytb synonymous diversity per genus (n = 46, R^2 ^= 0.10, p = 0.02, Figure 7). These analyses confirm that the mutation rate is a major determinant of mtDNA polymorphism in birds as well as in mammals.

Finally, we compared the ratio of non-synonymous to synonymous changes within (*P*_*n*_/*P*_*s*_) and between species (*D*_*n*_/*D*_*s*_) in birds and mammals – these ratio were computed when an outgroup was available. The two groups showed a similar *D*_*n*_/*D*_*s *_(birds: n = 81, average *D*_*n*_/*D*_*s *_= 0.019 ± 0.011; mammals: n = 76, average *D*_*n*_/*D*_*s *_= 0.023 ± 0.024; R^2 ^= 0.01, p = 0.11), but the *P*_*n*_/*P*_*s *_ratio was higher in birds than in mammals (birds: average *P*_*n*_/*P*_*s *_= 0.118 ± 0.110; mammals: average *P*_*n*_/*P*_*s *_= 0.076 ± 0.055; R^2 ^= 0.05, p < 0.01), as previously reported by Berlin *et al*. [24].

## Discussion

### mtDNA substitution rates – support for the longevity hypothesis

We showed that the mtDNA mutation rate, measured through the neutrally-evolving third codon positions of cytb, is highly variable between bird lineages. The molecular clock hypothesis does not apply to bird mtDNA evolution, fast-evolving species being thirty times more rapid than slow-evolving ones. The method we applied is particularly prone to reveal the actual amplitude of substitution rate variation: by overcoming the problem of mutational saturation, it allowed us to use an extensive dataset encompassing the whole taxonomic diversity of birds. This study, therefore, provides a synthetic view of substitution rate variations across bird lineages, bringing an important result in the field of molecular dating, in which the rough approximation of 2% of substitution per million of years is still debated [41,40,43,39]. Our analysis suggests that the molecular clock assumption should be avoided as far as bird mtDNA is concerned. The conclusions of Pereira and Baker [43] (obtained from full-genome data), and some studies done at smaller scale (reviewed in [39]) are here confirmed by cytochrome *b *third codon positions at wide taxonomic scale. Although the average rate in birds is close to this value, the 2% per site per million year calibration is a very bad summary of the whole picture: the rate is essentially (up to five times) higher than 2% in passerines, and (up to ten times) lower in non-passerines (Figure 1). Users of mtDNA as a tools for inferring divergence dates should imperatively use statistical phylogenetic methods accounting for substitution rate variation across lineages, the so-called clock-relaxed methods [44,48-50].

Several interesting results were revealed thanks to the comparison with mammals. First, birds show a more narrow range of variation of life-history traits than mammals. For example body mass varies from 5.5 g to 3.9 kg in the bird dataset, whereas it varies from 3.7 g to 138 tones in mammals. This result confirms the general influence of life-history traits on neutral mtDNA substitution rate in birds and mammals. This result is important because such a relationship was not reported in every taxonomic group [51], and even not by some studies in mammals [52,53]. Secondly, the neutral mtDNA substitution rate is lower in birds than in mammals, and this difference increases if body mass is regressed out of the analysis (Table 2). For example, birds evolve four times slower than mammals as far as small species (body mass < 500 g) are concerned. This result is in agreement with the longevity hypothesis, but not with the metabolic rate hypothesis. According to the latter model, birds should show a higher average mutation rate than mammals, because of their higher mass-specific metabolic rate and their lower average body mass [54].

During the course of evolution, birds have acquired adaptations to manage their high mass-specific metabolic rate, including an increased of reactive oxygen species (ROS) protection and lower ROS production [55,56]. In the context of the mitochondrial theory of aging, which postulates that ROS production as a byproduct of mitochondrial respiration contributes to aging, these adaptations could be interpreted as a response to the elevated longevity of birds [57]. The reduced rate of ROS production could explain the lower average mutation rate of birds mtDNA, as compared to mammals (see also [26]). It is surprising, therefore, to find a predominant influence of body mass, not longevity, in the within-birds analysis. Passerines show a positive relationship between longevity (or body mass) and substitution rate, which is quite surprising. We have no valid explanation for this relationship so far, perhaps because it is not biologically relevant – this relationship is the single result that was not recovered when we used other methods of substitution rate estimation (see Additional file 3). Within non-passerines, which are essentially long-lived species, body mass is still the variable best explaining substitution rate variations (Table 2). Using DNA-DNA hybridization data (and a smaller dataset), Mooers and Harvey [32] found no support for the metabolic rate hypothesis, but reported a significant generation time effect. In this context, it would be interesting to enlarge the female sexual maturity data set, in order to test the generation time hypothesis with our extended mitochondrial dataset.

### Determination of mtDNA diversity in birds

The neutral genetic diversity primarily depends on effective population size and mutation rate. Because population sizes appear so variable in time and between species, variations in genetic diversity across species have been frequently interpreted in demographic terms, *e.g*. [58,59]. Recently, the effect of population size on mtDNA diversity has been challenged [18,20]. The large variations in mutation rates we report in birds suggest that mutational effects should be carefully taken into account in studying mtDNA polymorphism in this group, as well as in mammals [18].

In general, the relationship between population size and mtDNA diversity is difficult to evaluate because of the scarcity of population size estimates for wild species. Life-history traits, like body mass, have therefore been used as proxy of population size [18,60]. Birds are an exception: thanks to the broad interest of the scientific community in bird ecology and systematics, direct estimates of species effective sizes are available for North American species [22]. We correlated these population size estimates to synonymous mtDNA diversity and found no significant relationship. The data set is rather small (n = 28), but we note that this relationship should theoretically be strong, given the wide range of population sizes (2 orders of magnitude). When using body mass as an indicator of population size, thus increasing much the data set, we found a negative correlation with cytb synonymous diversity, but this result was not significant. Berlin *et al*. [24] report a significant relationship between *π*_*s *_and body mass in birds using genus averages. If we apply the same method, we also find a significant relationship (n = 37, R^2 ^= 0.16, *p *= 0.01), but the effect is removed when the passerine/non-passerine status is taken into account (multiple regression, *p *body mass = 0.25). This result is in agreement with our previous mammalian analysis, in which the weak effect of body mass was removed by a taxonomic control [18]. So similarly to mammals, the mitochondrial genetic diversity is essentially uncorrelated to life-history traits in birds, and when it is so, it is most likely via the influence of mutation rate, not population size.

### Mitochondrial selective regime in birds

To explain the lack of relationship between mtDNA diversity and proxies of population size in mammals, Nabholz *et al*. [18] invoked demographic stochasticity, while Bazin *et al*. [20] invoked recurrent hitchhiking effects for other species having larger expected population sizes (invertebrates and marine species). What about Birds? To address this question we computed the neutrality index (NI = *P*_*n*_/*P*_*s*_/*D*_*n*_/*D*_*s*_; [61], see Methods). NI < 1 indicates positive selection, while NI > 1 indicates purifying selection. The average NI in birds is significantly higher than in mammals (R^2 ^= 0.058, p < 0.01, Additional file 2) and only three species of birds show a NI < 1 (seven in mammals). The high NI values in birds are due to their high *P*_*n*_/*P*_*s *_ratios -*D*_*n*_/*D*_*s *_ratios are similar to those of mammals. This result indicates that mtDNA evolution in birds is mainly governed by purifying selection, as previously reported [24]. It is also indicative of a strong level of constraint acting on bird cytochrome *b *sequences, which is known as the avian constraint hypothesis [62].

Like mammals, birds appear to have population sizes low enough to belong to the "drift domain", rather than the "draft domain" *sensu *Gillespie [63] (see discussion in [18]). In the absence of a significant impact of positive selection, we have to invoke strong demographic stochasticity to explain the lack of relationship between population size and mtDNA diversity. Mutation rate is expected to be a major determinant of within-species genetic diversity whether populations are at mutation-drift equilibrium (standard theory) or not [64,65]. Consistently, we report a significantly positive relationship between cytb diversity and substitution rate (Figure 7), again paralleling the mammalian situation.

In a recent paper, Berlin *et al*. [24] proposed an alternative hypothesis to explain mtDNA polymorphism patterns in birds. They suggested that Hill-Robertson effects due to complete association with the W chromosome could explain the lower synonymous mitochondrial diversity and the higher *P*_*n*_/*P*_*s *_observed in birds, as compared to mammals. This should also explain the lack of correlation between neutral polymorphism and population size. Outwardly, we confirm this hypothesis by showing that the lower synonymous mitochondrial diversity is not found for nuclear allozymes. But this picture is complicated by the globally lower mtDNA substitution rate in birds than in mammals. Controlling for mutation rates actually removes the major part of the difference between birds and mammals. To quantify the effect of mutation rate variation on synonymous mtDNA diversity we combined the bird and mammal polymorphism and neutral substitution rate datasets and performed a multiple regression of substitution rate and bird/mammal status (class) on synonymous mtDNA diversity. This model explains 21% of *π*_*s *_variation (birds: n = 46, mammals: n = 123, *p *< 0.001). The result also shows that the mutation rate has a strong effect on *π*_*s *_(t = 4.9; *p *< 0.001) and considerably reduces the effect of class (t = 2.1, *p *= 0.021). This analysis indicates that a subtential part of the difference between birds and mammalian *π*_*s *_is explained by their distinct mutation rates (see Additional file 4b). The difference in mutation rates, however, cannot explain the higher level of mitochondrial *P*_*n*_/*P*_*s *_found in birds. Birds and mammals have apparently comparable effective population sizes, as suggested by their similar average allozymic heterozygosity. The higher *P*_*n*_/*P*_*s *_ratio in birds may therefore be due to Hill-Robertson interferences between the mitochondrial and W chromosomes, as suggested by Berlin *et al*. [24], although this effect has only a weak influence on absolute levels of *π*_*s*_, when mutation rate is controlled for.

## Conclusion

In this work, we achieved a comprehensive comparison of mitochondrial substitution rate variation and mitochondrial diversity between birds and mammals. Cytb neutral substitution rates are, on average, 3.9 times slower in birds than in mammals. Among-lineages variability, although substantial, is also lower in birds than in mammals. This analysis corroborates the longevity hypothesis about mutation rate determination [14,17]. Despite their higher metabolic rate, birds undergo a lower mitochondrial mutation rate, probably because of the beneficial effect of reduced oxidative damage in long-lived species [26,28]. We suggest that the low mtDNA mutation rate of birds might explain their exceptional mass-specific longevity.

As for mammals, we report no evidence of adaptive selection on cytb sequence evolution, but still no population size effect, even when using direct estimates of population size available for North American species. We thus invoke the same demographic instability explanation as previously proposed for mammals [18], although selective interferences with the W chromosome [24] could contribute to the noisy relationship between mtDNA diversity and population size. We also confirm the importance of mutation rate as a determinant of mtDNA diversity – the lower mutation rates in birds almost fully explain their lower synonymous diversity.

This study confirms and extends the message of caution expressed by Nabholz *et al*. [18,14] about the usage of mtDNA as a molecular marker of biodiversity in vertebrates: (i) mtDNA diversity is not related to species abundance; (ii) mtDNA greatly departs the molecular clock hypothesis. The 2% per site per million year calibration (estimated from primate data) has no degree of generality, and should not be used for dating purposes in the absence of fossil data.

## Methods

### Sequence data for substitution rate analysis

Complete bird cytochrome *b *sequences were extracted from National Center for Biotechnology information/Genbank. One sequence per species (the longest, excluding indeterminations) was selected.

Accession numbers are given in Additional file 5. The 5'-most 102 nucleotides were removed from the alignments, because missing in a large number of species. Total alignment length was 1043 nucleotides.

### Sequence data and Allozyme data for polymorphism analysis

An mtDNA dataset was built from Polymorphix [46]. Polymorphix is a database dedicated to sequence polymorphism. It contains within-species homologous sequence families built from EMBL/GenBank under suitable similarity and bibliographic criteria. To obtain a homogeneous dataset, comparable with a previous mammalian study [18], only the protein coding cytochrome *b *gene was used. We extracted from Polymorphix every bird sequence family for which any >100-bases-long cytochrome *b *fragment was available in four individuals or more. Polymorphix sequence families were aligned using CLUSTALW [66], inspected by eye and corrected when required. Dubious sequences (badly aligned or including many undetermined nucleotides) were manually removed. Alignments are available at .

Estimates of allozyme heterozygosity in 74 bird and 194 mammalian species were obtained from [47]. Allozyme heterozygosities were averaged using 10–30 loci. The most popular allozyme loci are shared by many species. Allozyme heterozygosities are therefore fairly comparable between species.

### Substitution Rate estimation

We used the same uncoupled method as previously developed for mammals [14]. The whole cytochrome *b *data set was split in groups within which sequence divergence is moderate. To achieve this, the GenBank taxonomic classification was traversed recursively, starting from birds orders and moving toward lower levels. For each traversed taxonomic group, we 1) gathered the corresponding third codon position sequences, 2) aligned sequences using ClustalW [66], 3) built a maximum likelihood phylogenetic tree using PHYML [67], general time reversible + Gamma model of nucleotide evolution, and 4) calculated the pairwise patristic distances for every pair of species (defined as the sum of branch lengths in the path connecting the 2 species in the tree). When the median pairwise distance between species was lower than 0.4, the current taxonomic group was selected for further analysis, (except for the Falconiformes and Ciconiiformes orders, for which monophyly is uncertain [68,69]), and the taxonomic traversal was stopped. Otherwise, the above procedure was applied to underlying taxonomic groups. The within group, species-specific relative neutral substitution rates were estimated using two different softwares: MULTIDIVTIME [44] and MCMCTREE [50,70] available in the PAML packages [71]. The major difference is that MULTIDIVTIME makes use of a normal approximation of the likelihood, and only implements the F84 + Γ model of sequence evolution [44], whereas MCMCTREE performs exact likelihood calculation and can be used with different models of sequence evolution – we used the HKY + Γ The Monte Carlo Markov Chain was run for 1 million generations after a burn-in of 200,000 generations that achieved stationarity.

Bird phylogeny is still partly uncertain, particularly the basal relationships within the Neoaves clade [68,69,72-74]. We used two alternative phylogenetic trees to date divergences. The first one is conform to Ericson *et al*. [68] topology, obtained using mostly nuclear markers, and the second one is conform to the complete genome mitochondrial topology proposed by Slack *et al*. [69]. In both topologies, the relationships within passerines were made congruent to Barker *et al*. [75]. Groups were dated by applying MULTIDIVTIME to amino-acid sequences (two representative species per group, model mtREV + G). Twelve fossil calibrations points were used (Additional file 6). The results were very similar whatever the method and the tree (Additional files 3 and 7), so we chose to present only the results obtained with MULTIDIVTIME using the mitochondrial genome topology, except when mentioned. We checked that neither the mean nor the variance of species-specific estimated substitution rates are correlated to terminal branch lengths (not shown). Sequence management and GenBank taxonomic exploration were achieved using homemade C++ programs based on the Bio++ libraries [76].

### Polymorphism sequence data analyses

Two measures of molecular genetic diversity were used, namely the nucleotide diversity *π *[77] and Watterson's statistics *θ*_*w *_[78]. In the case of the haploid, maternally transmitted mtDNA, both statistics are unbiased estimates of the *N e f μ *product under the assumption of neutrality and mutation/drift equilibrium, where *N e f *is the effective population size of females and *μ *the locus mutation rate. *π *and *θ*_*w *_were calculated from the total length of the analysed fragments, and expressed in per-site level of diversity (after being divided by sequence length). We also measured the synonymous and non-synonymous nucleotide diversity (*π*_*s*_/*π*_*n*_), also expressed per synonymous site and per non-synonymous site.

The Neutrality Index (NI, [61]) was calculated for dataset 1 when outgroups were available. This index aims at comparing the ratio of non-synonymous (= amino acid changing) to synonymous (silent) changes within species (*π*_*n*_/*π*_*s*_) and between species (*D*_*n*_/*D*_*s*_): NI is one when evolution is neutral, higher than one under purifying selection, and lower than one in case of adaptation. *π*_*n *_and *π*_*s *_were estimated as described above. *D*_*n *_and *D*_*s *_are the non-synonymous and synonymous pairwise sequence divergences between related taxa, calculated using the maximum likelihood method implemented in codeml in PAML 4 [71]. Excluding low frequency variants (<0.125) when estimating NI yielded similar results (not shown). The phylogenetic tree was obtain using PHYML software [67] with TN93 + Γ model of sequences evolution. We used the maximum likelihood method because of the potential underestimation of *D*_*s *_in simple pairwise distance due to multiple hits. Alignments are available at .

### Life History data and Population size estimation

Body mass, age of female sexual maturity, and maximum longevity were obtained from the AnAge database [79]. Direct estimates of population size are available for North American breeding birds in the North American Landbird Conservation Plan, Breeding Bird Survey abundance data [22], . Life history data and rate estimates are available at .

### Statistical analyses

Genetic diversity measures were arcsine-transformed [80] and analyzed under the general linear model assumptions using R [81]. Quantitative life-history variables were log-transformed. One-way and two-way ANOVA with interactions were performed on transformed variables. Fisher's tests were performed using class II errors.

## Authors' contributions

All authors designed research project. BN compiled the data and performed the analyses. All authors wrote and approved the final manuscript.

## Supplementary Material

Additional file 1 **Figure S1**. Distribution of bird maximum longevity. The maximum longevity is log transformed and is in years. Black: whole dataset; shading yellow: passerines birds (n = 88); shading blue: no passerines birds (n = 108) Click here for file

Additional file 2 **Figure S2**. Neutrality index (NI) distribution in birds (n = 81) and mammals (n = 75). NI values greater than 20 were forced to 20 for clarity. Click here for file

Additional file 3 **Table S4**. Effects of life-history variables on mtDNA substitution rate in passerines versus other bird species according the different combination of topologies and programs. Click here for file

Additional file 4 **Figure S3**. a) mtDNA synonymous diversity (*π*_*s*_) in birds (n = 46) and mammals (n = 123). The median, quartiles, and extrema of the distribution of *π*_*s *_are given. b) Residuals of the relationship between mtDNA synonymous diversity (*π*_*s*_) and third-codon position substitution rate in birds and mammals. Click here for file

Additional file 5 **Table S2**. GenBank accession numbers, taxonomy, mitochondrial substitution rate and life-history traits of the 1571 species used in the substitution rate analysis. Click here for file

Additional file 6 **Table S1**. Fossil calibration dates used in this study (in million years). Click here for file

Additional file 7 **Table S3**. Effects of life-history variables on mtDNA substitution rate in bird species according the different combination of topologies and programs. Click here for file
