# Supplementary figures and images for: The erratic mitochondrial clock: variations of mutation rate, not population size, affect mtDNA diversity across birds and mammals
Source: BMC Evol Biol. 2009 Mar 10;9:54. doi: 10.1186/1471-2148-9-54 (PMC2660308; doi:10.1186/1471-2148-9-54)

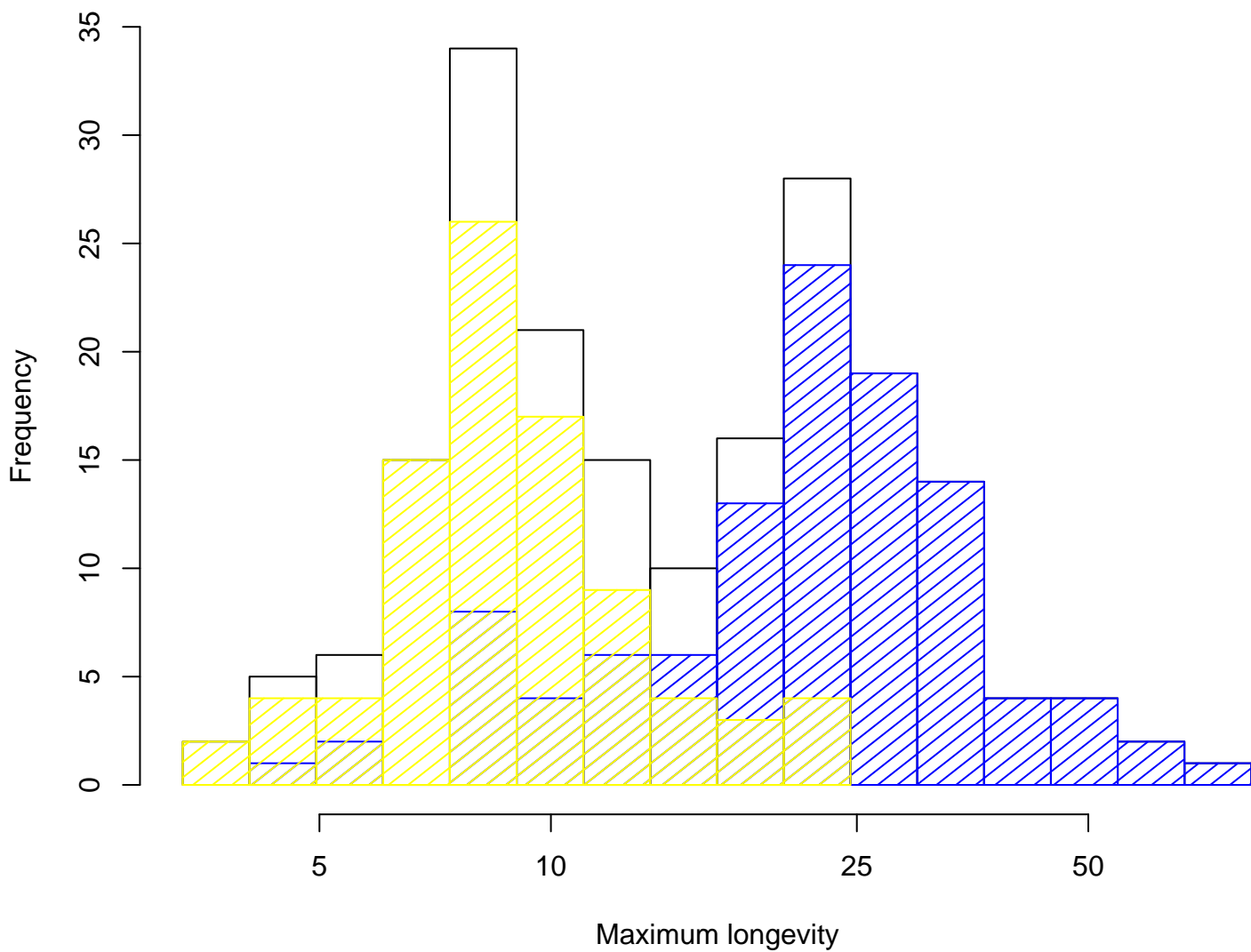

Supplement: Additional file 1 — Figure S1. Distribution of bird maximum longevity. The maximum longevity is log transformed and is in years. Black: whole dataset; shading yellow: passerines birds (n = 88); shading blue: no passerines birds (n = 108) [file 1471-2148-9-54-S1.pdf]

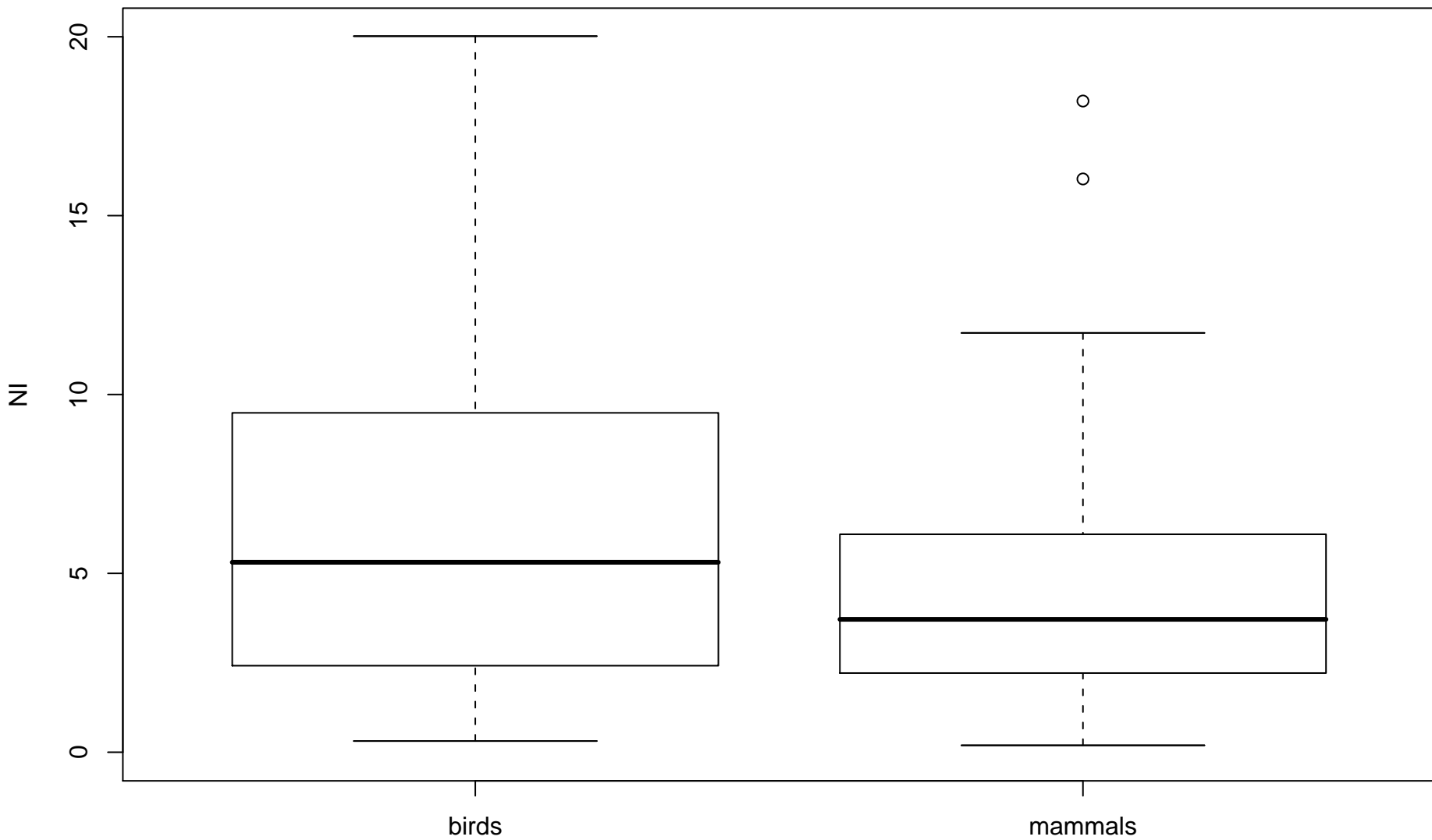

Supplement: Additional file 2 — Figure S2. Neutrality index (NI) distribution in birds (n = 81) and mammals (n = 75). NI values greater than 20 were forced to 20 for clarity. [file 1471-2148-9-54-S2.pdf]

Synonymous Nucleotide diversity

**a**

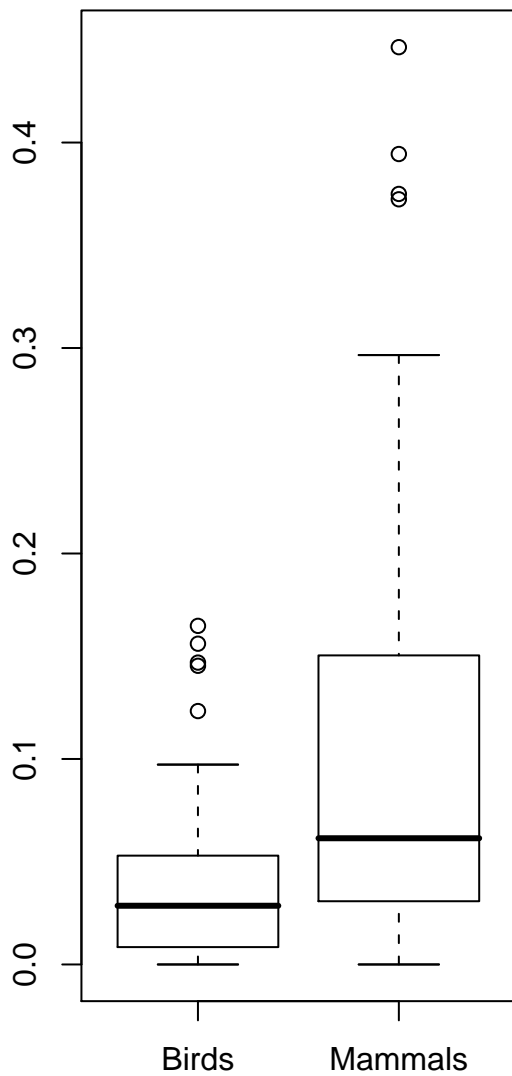

**b**

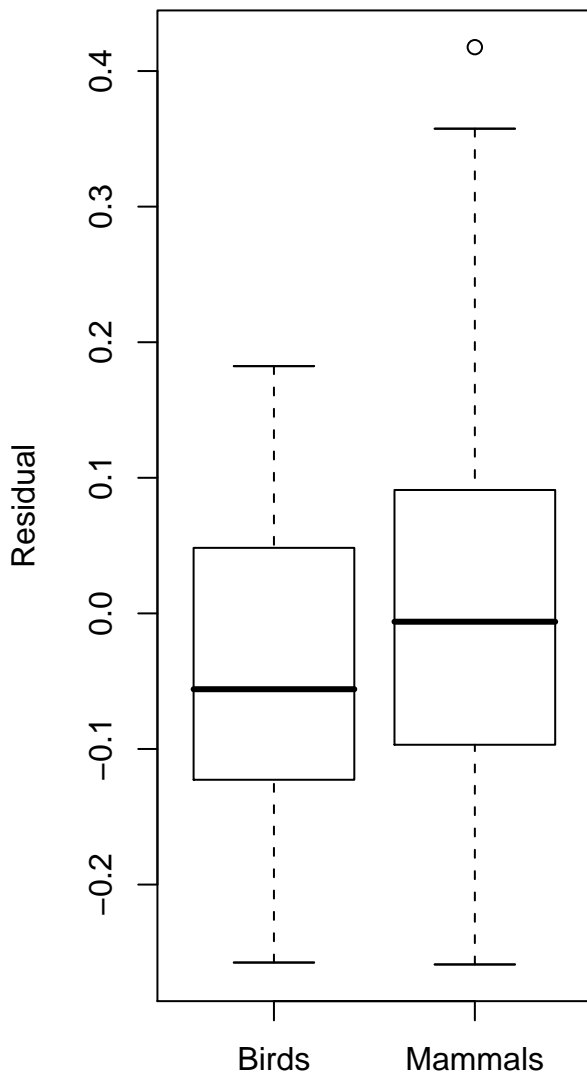

Supplement: Additional file 4 — Figure S3. a) mtDNA synonymous diversity (πs) in birds (n = 46) and mammals (n = 123). The median, quartiles, and extrema of the distribution of πs are given. b) Residuals of the relationship between mtDNA synonymous diversity (πs) and third-codon position substitution rate in birds and mammals. [file 1471-2148-9-54-S4.pdf]
