# Supplementary material for: The erratic mitochondrial clock: variations of mutation rate, not population size, affect mtDNA diversity across birds and mammals
Source: BMC Evol Biol. 2009 Mar 10;9:54. doi: 10.1186/1471-2148-9-54 (PMC2660308; doi:10.1186/1471-2148-9-54)
Supplement: Additional file 3 — Table S4. Effects of life-history variables on mtDNA substitution rate in passerines versus other bird species according the different combination of topologies and programs. [file 1471-2148-9-54-S3.pdf]

Table S4 - Effects of life-history variables on mtDNA substitution rate in passerines versus other bird species according the different combination of topologies and programs.

		Passerines (n=88)				Non-passerines (n=108)			
		slope	R^2	*p*1	*p*2	slope	R^2	*p*1	*p*2
MULTIDIVTIME & Ericson topology	Body mass	0.22	0.12	**<0.01**		-0.15	0.09	**<0.01**	
	Maximum longevity	0.48	0.15	**<0.01**		-0.37	0.07	**0.01**	
	Body mass + Maximum longevity		0.17	0.13	**0.02**		0.11	**0.04**	0.13
MCMCTREE & Mitochondrial topology	Body mass	-0.24	0.11	**<0.01**		-0.10	0.14	**<0.01**	
	Maximum longevity	-0.29	0.04	**0.05**		-0.25	0.11	**<0.01**	
	Body mass + Maximum longevity		0.11	**0.01**	0.79		0.17	**<0.01**	0.06
MCMCTREE & Ericson topology	Body mass	-0.25	0.11	**<0.01**		-0.08	0.07	**<0.01**	
	Maximum longevity	-0.30	0.05	**0.05**		-0.20	0.06	**0.01**	
	Body mass + Maximum longevity		0.11	**0.01**	0.77		0.09	0.07	0.18
