# Supplementary material for: The erratic mitochondrial clock: variations of mutation rate, not population size, affect mtDNA diversity across birds and mammals
Source: BMC Evol Biol. 2009 Mar 10;9:54. doi: 10.1186/1471-2148-9-54 (PMC2660308; doi:10.1186/1471-2148-9-54)
Supplement: Additional file 6 — Table S1. Fossil calibration dates used in this study (in million years). [file 1471-2148-9-54-S6.pdf]

Divergence	Maximal Date	Minimal Date	Used in analysis**	Reference
Stem Piciformes	Free	32	Slack / Ericson	Ericson *et al.* 2007
Stem Trogontiformes	Free	53	Slack / Ericson	Ericson *et al.* 2007
Coliiformes	Free	55	Slack / Ericson	Ericson *et al.* 2007
Strigiformes	Free	60	Slack / Ericson	Ericson *et al.* 2007
Spheniscidae / Ciconidae	72	62	Slack	Slack *et al.* 2006
Apodiformes	Free	53	Slack / Ericson	Ericson *et al.* 2007
Trochilidae	Free	47.5	Slack / Ericson	Ericson *et al.* 2007
Anseranas / Anas	72	66	Slack / Ericson	Slack *et al.* 2006<br>Clarke *et al.* 2005
Galliformes	Free	53	Slack / Ericson	Ericson *et al.* 2007
Gaviiformes	Free	32	Slack / Ericson	Ericson *et al.* 2007
Oscine / Suboscine (Passerine)	77*	55	Slack / Ericson	Ericson *et al.* 2002,<br>Barker *et al.* 2004<br>Boles 1995
Stem Spheniscidae	Free	60	Ericson	Ericson *et al.* 2007

*This calibration is secondary points based on Gondwana time frame for passerine evolution.

** We used two alternative phylogenetic trees to date divergences. The first one is conform to Ericson *et al.* 2006 topology and the second one is conform to topology proposed by Slack *et al.* 2006.

Barker FK, Cibois A, Schikler P, Feinstein J, Cracraft J: **Phylogeny and diversification of the largest avian radiation.** *Proceedings of the National Academy of Sciences of the United States of America* 2004, 101: 11040-11045

Boles W: **The World's oldest songbirds.** *Nature* 1995, 374: 21-22

Clarke JA, Tambussi CP, Noriega JI, Erickson GM , Ketcham RA: **Definitive fossil evidence for the extant avian radiation in the Cretaceous.** *Nature* 2005, 433: 305-3088

Ericson PGP, Anderson CL, Britton T, Elzanowski A, Johansson US, Kallersjo M, Ohlson JI, Parsons TJ, Zuccon D, Mayr G: **Diversification of neoaves: integration of molecular sequence data and fossils.** *Biology Letters* 2006, 2: 543-5U1

Ericson PGP, Christidis L, Cooper A, Irestedt M, Jackson J, Johansson US, Norman JA: **A Gondwanan origin of passerine birds supported by DNA sequences of the endemic New Zealand wrens.** *Proc Biol Sci* 2002, 269: 235-241

Slack KE, Jones CM, Ando T, Harrison GLA, Fordyce RE, Arnason U, Penny D: **Early penguin fossils, plus mitochondrial genomes, calibrate avian evolution.** *Molecular Biology and Evolution* 2006, 23: 1144-1155
