# Supplementary material for: The erratic mitochondrial clock: variations of mutation rate, not population size, affect mtDNA diversity across birds and mammals
Source: BMC Evol Biol. 2009 Mar 10;9:54. doi: 10.1186/1471-2148-9-54 (PMC2660308; doi:10.1186/1471-2148-9-54)
Supplement: Additional file 7 — Table S3. Effects of life-history variables on mtDNA substitution rate in bird species according the different combination of topologies and programs. [file 1471-2148-9-54-S7.pdf]

Table S3 - Effects of life-history variables on mtDNA substitution rate in bird species according the different combination of topologies and programs.

	Models	Without Phylogenetic control			
		slope	R^2	*p*1	*p*2
MULTIDIVTIME & Ericson topology	Body mass	-0.25	0.37	**<0.01**	
	Maximum longevity	-0.66	0.24	**<0.01**	
	Body mass + Maximum longevity		0.37	**<0.01**	0.46
MCMCTREE & Mitochondrial topology	Body mass	-0.28	0.57	**<0.01**	
	Maximum longevity	-0.78	0.42	**<0.01**	
	Body mass + Maximum longevity		0.59	**<0.01**	**0.01**
MCMCTREE & Ericson topology	Body mass	-0.28	0.54	**<0.01**	
	Maximum longevity	-0.79	0.39	**<0.01**	
	Body mass + Maximum longevity		0.55	**<0.01**	**0.03**
